# Consensus on Upper Gastrointestinal Endoscopy Key Performance Indicators to Reduce Post Endoscopy Upper Gastrointestinal Cancer

**DOI:** 10.1002/ueg2.70001

**Published:** 2025-07-24

**Authors:** Umair Kamran, Toto Anne Gronlund, Eva J. A. Morris, Matthew Brookes, Matt Rutter, Mimi McCord, Nicola J. Adderley, Nigel Trudgill

**Affiliations:** ^1^ Department of Gastroenterology Sandwell and West Birmingham NHS Trust Birmingham UK; ^2^ James Lind Alliance Southampton UK; ^3^ Nuffield Department of Population Health Big Data Institute University of Oxford Oxford UK; ^4^ Department of Gastroenterology The Royal Wolverhampton NHS Trust Wolverhampton UK; ^5^ Faculty of Science and Engineering Research Institute in Healthcare Science University of Wolverhampton Wolverhampton UK; ^6^ Department of Gastroenterology North Tees and Hartlepool NHS Foundation Trust Hartlepool UK; ^7^ Faculty of Medical Sciences Population Health Sciences Institute Newcastle University Newcastle UK; ^8^ Heartburn Cancer UK Southampton UK; ^9^ Institute of Applied Health Research University of Birmingham Birmingham UK; ^10^ Institute of Cancer and Genomic Sciences University of Birmingham Birmingham UK

**Keywords:** England, gastrointestinal cancer, key performance indicators, standardization, training, upper gastrointestinal endoscopy

## Abstract

**Background:**

Upper gastrointestinal (UGI) endoscopy lacks established key performance indicators. Up to three‐fold variation in post endoscopy upper gastrointestinal cancer rates has been observed among endoscopy providers in England, highlighting the need for standardisation of UGI endoscopy practices.

**Objective:**

We aimed to achieve consensus on evidence‐based key performance indicators to reduce post endoscopy upper gastrointestinal cancer.

**Methods:**

Modified nominal group technique was employed in two consensus workshops, with representation from clinicians, patients and relatives, moderated by James Lind Alliance facilitators. Potential indicators were identified from the umbrella systematic review, English provider post endoscopy upper gastrointestinal cancer rates, and differences in endoscopy practices from the National Endoscopy Database between providers with the highest (worst) and lowest (best) post endoscopy upper gastrointestinal cancer rates. KPIs were categorised as provider or endoscopist/procedure related and ranked as of major or minor importance. Minimum standards were proposed where possible.

**Results:**

Participants included 14 clinicians (gastroenterologists and UGI surgeons), 3 nurse endoscopists, 2 UGI cancer nurse specialists, 14 patients, their relatives and representatives from patient support groups and four observers.

Endoscopy provider related major key performance indicators and proposed standards included monitoring post endoscopy upper gastrointestinal cancer rates (minimum standard ≤ 7%); less intense endoscopy lists (maximum 10 ‘points’ per list [one point is equivalent to 15 min]); endoscopy provider accreditation (all providers); and premalignant condition surveillance on dedicated lists by endoscopists with adequate training (> 90% surveillance endoscopies).

Endoscopist/procedure related major key performance indicators included: examination time ≥ 7 min; training in early UGI neoplasia recognition (all endoscopists); mucosal view quality recorded and cleansing agents used if not excellent (> 90% endoscopies); intravenous sedation offered to all appropriate patients; recommended number of biopsies from cancer associated or premalignant lesions (> 90% endoscopy where such lesions identified); and endoscopists' annual UGI endoscopy volume > 100 (all endoscopists).

**Conclusion:**

This study offers a consensus on the key performance indicators and minimum standards that should be used to improve UGI endoscopy quality and reduce post endoscopy upper gastrointestinal cancer.

## Graphical abstract

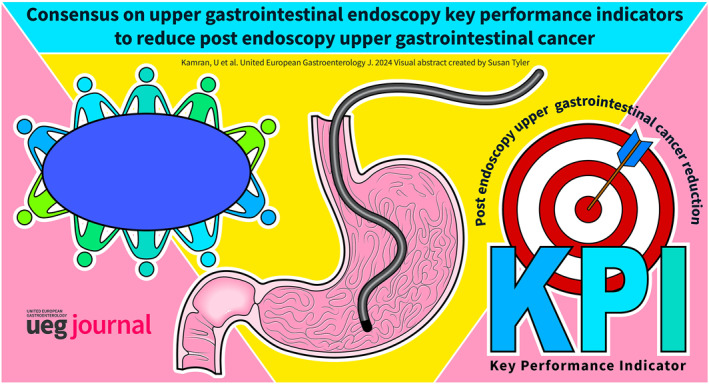

1

**Summary**

- Summarize the established knowledge on this subject
  ◦ Upper gastrointestinal (UGI) endoscopy lacks established key performance indicators (KPIs).
  ◦ Up to three fold variation in the post endoscopy UGI cancer (PEUGIC) rate has been reported among endoscopy providers in England, indicating significant variation in the quality of UGI endoscopy.
  ◦ Endoscopy societies have proposed a number of recommendations, mainly focussing on the general approach to performing UGI endoscopy.
- What are the significant and/or new findings of this study?
  ◦ The current study employed a modified nominal group technique to reach consensus on the KPIs for UGI endoscopy to reduce PEUGIC.
  ◦ Endoscopy provider related major KPIs included monitoring of PEUGIC rates, less intense endoscopy lists, external accreditation and premalignant condition surveillance on dedicated lists by endoscopists with adequate training.
  ◦ Endoscopist or procedure related major KPIs included adequate examination time, training in early UGI neoplasia recognition, use of cleansing agents if mucosal views not excellent, offering intravenous sedation to all appropriate patients, recommended number of biopsies from cancer associated or premalignant lesions and endoscopists' annual UGI endoscopy volume.

## 2 Introduction

Upper gastrointestinal (UGI) endoscopy is considered the gold standard investigation to diagnose UGI cancers, and associated premalignant conditions, mainly located in the oesophagus and stomach. 1.2 million UGI endoscopies are performed and approximately 16,500 people are diagnosed with UGI cancer each year in the UK [1, 2]. However, more than 8% of patients with UGI cancer in England had an endoscopy which failed to detect cancer in the 3 years before cancer diagnosis, termed post endoscopy UGI cancer (PEUGIC) [3]. Given that the earlier cancers are diagnosed the better the survival outcomes, reducing the rates of PEUGIC is a priority. Therefore, it is imperative to ensure the highest standards and quality in endoscopy to ensure UGI cancers are diagnosed at the earliest stage, or even at a premalignant stage, and thus reduce PEUGIC.

The Joint Advisory Group (JAG) on gastrointestinal endoscopy oversees the certification for and assessment of competency in the performance of UGI endoscopy in the UK and the main focus is the technical competence and safety of the procedure. Endoscopy societies, including the British Society of Gastroenterology (BSG), the European Society of Gastrointestinal Endoscopy (ESGE) and the American Gastroenterological Association (AGA), have proposed UGI endoscopy quality standards, but these are mainly focussed on the general approach to diagnostic endoscopy [4, 5, 6]. An Asian statement on the endoscopy quality standards to detect early upper gastrointestinal (UGI) neoplasia was published in 2019; however, the quality of evidence for most of the standards was poor and based on expert opinion [7].

## 3 Aims and Scope

We aimed to develop consensus on key performance indicators (KPI) to be used to enhance the quality of UGI endoscopy diagnosis and reduce the risk of PEUGIC.

The consensus process focussed on the following domains:

- Identification of potential KPIs
- Ranking of KPIs as major and minor quality indicators
- Suggestion of minimum target standards where possible

The following aspects were not included:

- Therapeutic endoscopy
- Transnasal endoscopy

## 4 Materials and Methods

### 4.1 Evidence Synthesis

Potential KPIs for UGI endoscopy were identified from the following resources:

#### 4.1.1 Systematic Review

Bibliographic databases were searched up to December 2021 and evidence for endoscopy and endoscopist related factors associated with the detection of dysplasia and UGI cancer was summarised [8].

#### 4.1.2 National Cancer Registry and Hospital Episode Statistics Database Analysis

Using linked National Cancer Registry and Analysis Services (NCRAS) and Hospital Episode Statistics (HES) databases, data for 98,801 patients were analysed who were diagnosed with UGI cancer within 3 years of an endoscopy without a cancer diagnosis in England between 2009 and 2018. The variation in PEUGIC rates among endoscopy providers in England was examined [3].

#### 4.1.3 National Endoscopy Database Analysis

Using the UK National Endoscopy Database (NED), endoscopy practices were compared between providers with the best and worst PEUGIC rates in England and endoscopy practices associated with the providers with the best PEUGIC rates identified.

KPIs were categorised as endoscopist/procedure related and endoscopy provider related. Endoscopy providers include both government (National Health Service) and private hospitals and facilities which offer endoscopy services to patients. Minimum standards were proposed based on either the evidence identified in the above resources or the expert opinion of the panel. Minimum standards for some of the KPIs could not be suggested due to insufficient data.

### 4.2 Consensus Workshops

We employed the modified Nominal Group Technique to develop consensus on the KPIs. This rigorous, yet flexible approach facilitates the generation and prioritisation of ideas from a diverse group of participants, ensuring a democratic and inclusive process [9]. This was conducted in the form of two online workshops moderated by James Lind Alliance facilitators. The James Lind Alliance is a non‐profit making initiative dedicated to bringing together clinicians, patients and carers to discuss research priorities. The National Institute for Health and Care Research funds their infrastructure.

In each workshop, the participants were divided into three groups with balanced clinician and patient/relative representation. A summary of the evidence and list of potential KPIs and minimum standards were circulated to participants in advance. The first workshop focussed on identifying candidate KPIs. Participants were asked to provide their opinion on the list of potential KPIs. This was followed by a ranking exercise within each of the three groups and KPIs were ranked as major and minor. Additional KPIs were added to the list based on the participants' opinions. The facilitators provided a summary of ranking from each small group session. In the second workshop, participants confirmed the ranking of the KPIs based on their importance and a consensus was reached by combining the ranking scores. There was some movement of members of the small groups between workshops to ensure an adequate balance of clinicians and lay members in each group, as 15% of participants were unable to attend both workshops.

### 4.3 Ranking of Indicators

The ranking system prioritised KPIs based on their relative importance in reducing PEUGIC. Endoscopy provider and endoscopist/procedure related KPIs were ranked separately. Final ranking scores from the small group sessions in the second consensus workshop were combined and the geometric mean was calculated by multiplying the ranks together, and then taking the *n*th root, where ‘n’ was the number of groups. This produces a geometric average which is better at respecting outlier ranks. The ranking system was designed such that a lower score denotes a more important indicator. The top 10 KPIs were considered major indicators and the remaining ones minor. The level of evidence for each KPI is reported using GRADE methodology (Table 1) [10].

**TABLE 1:** The quality of evidence and definitions according to the grading of recommendations assessment, development and evaluation (GRADE) system.

Quality of evidence	Definition
High	Further research is very unlikely to change our confidence in the estimate of effect
Moderate	Further research is likely to have an important impact on our confidence in the estimate of effect and may change the estimate
Low	Further research is very likely to have an important impact on our confidence in the estimate of effect and is likely to change the estimate
Very low	Any estimate of effect is very uncertain

## 5 Results

### 5.1 Participants

Participants included 14 clinicians (consultant gastroenterologists, UGI surgeons and trainees), 3 nurse endoscopists, 2 UGI cancer nurse specialists, 8 patients, 3 carers, 3 representatives from patient support groups, and 4 observers.

### 5.2 Key Performance Indicators

A list of KPIs, proposed minimum standards and the quality of evidence for each is provided in Table 2. Mean ranking scores for each KPI are reported in Table 3 and Supporting Information S1.

**TABLE 2:** List of proposed key performance indicators for upper gastrointestinal endoscopy and their quality of evidence.

Quality indicators	Minimum standards	Quality of evidence
Major indicators		
Endoscopy provider related		
1. Monitoring of PEUGIC rates	≤ 7%	Moderate
2. Less intense endoscopy lists	< 10 points per list	Low
3. External accreditation of endoscopy providers	All providers	Low
4. Surveillance of high‐risk conditions performed on dedicated lists by endoscopists with appropriate training and adequate time allocated.	> 90% of surveillance UGI endoscopies	Moderate
Endoscopist or procedure related		
5. Adequate examination time	≥ 7 min	Moderate
6. Endoscopists should have dedicated training in the recognition of early UGI neoplasia	All endoscopists	Low
7. Quality of mucosal views recorded and mucosal cleaning agents employed if not excellent mucosal views	> 90% of diagnostic endoscopies	Moderate
8. Intravenous sedation offered to all appropriate patients	Unable to suggest	Moderate
9. If a cancer associated or premalignant lesion is identified, recommended number of biopsies taken	> 90% of endoscopies where a cancer associated, or premalignant lesion is identified	Low
10. Endoscopists' annual upper gastrointestinal endoscopy volume	> 100 upper GI endoscopies/year	Moderate
Minor indicators		
1. Detection rate for premalignant conditions	Unable to suggest	Low
2. Image enhancement techniques used especially in high‐risk patients	Unable to suggest	High
3. Photo documentation of important anatomical sites	> 90% of diagnostic upper GI endoscopies	Low
4. Neoplasia detection rate	Unable to suggest	Low
5. Artificial intelligence	Unable to suggest	Low

Abbreviation: PEUGIC, Post Endoscopy Upper Gastrointestinal Cancer.

**TABLE 3:** Mean ranking scores for the key performance indicators for upper gastrointestinal endoscopy.

Key performance indicators	Mean ranking score
Endoscopy provider related	
1. Monitoring of endoscopy provider PEUGIC rate	2.0
2. Less intense endoscopy lists	2.1
3. External accreditation of endoscopy providers	2.3
4. Surveillance of high‐risk conditions performed on dedicated lists by endoscopists with appropriate training and adequate time allocated	2.5
Endoscopist or procedure related	
5. Minimum examination time ≥ 7 min	2.0
6. Endoscopists should have dedicated training in recognition of early UGI neoplasia	2.2
7. Mucosal cleaning agents used to achieve good mucosal views	3.1
8. Intravenous sedation offered to all appropriate patients	4.0
9. If a cancer associated or premalignant lesion is identified, recommended number of biopsies taken	4.3
10. Minimum annual upper gastrointestinal endoscopy volume > 100	4.9
11. Detection rate for premalignant conditions monitored	7.2
12. Image enhancement techniques used, especially in high‐risk patients	9.6
13. Photo documentation of important anatomical sites	10.2
14. Neoplasia detection rate monitored	12.2
15. Artificial intelligence	13.7

Abbreviation: PEUGIC, Post Endoscopy Upper Gastrointestinal Cancer.

#### 5.2.1 Endoscopy Provider Related

Four provider related KPIs were identified, all ranked as major and included: monitoring of PEUGIC rates (minimum standard ≤ 7%); less intense endoscopy lists (less than 10 points per list ‐ in the UK 15 min are assigned for one point and a diagnostic UGI endoscopy is allocated one point); external accreditation of endoscopy providers (all providers); and surveillance of upper gastrointestinal premalignant conditions performed by endoscopists with appropriate training and adequate time allocated (> 90% of surveillance endoscopies). The level of evidence for these KPIs was graded as low to moderate. Monitoring of PEUGIC rates and less intense endoscopy lists were ranked as the most important KPIs.

#### 5.2.2 Endoscopist or Procedure Related

In total, 11 endoscopist or procedure related KPIs were identified, six of these ranked as major and included: adequate examination time (at least 7 min); endoscopists should have dedicated training in the recognition of early UGI neoplasia (all endoscopists); the quality of mucosal views recorded and mucosal cleansing agents employed if not excellent mucosal views (> 90% of diagnostic endoscopies); intravenous sedation offered to all appropriate patients (unable to suggest minimum standard); if a cancer associated or premalignant lesion is identified, the recommended number of biopsies taken (> 90% of endoscopy where a cancer associated or premalignant lesion identified); endoscopists' annual UGI endoscopy volume (> 100 UGI endoscopies/year). The level of evidence for these KPIs was graded as low to moderate. Adequate examination time and endoscopist training in lesion recognition were ranked as the most important KPIs by all three groups.

Five KPIs were ranked as minor and included: use of image enhancement techniques in high risk patients; photo documentation of important anatomical sites, neoplasia detection rate, detection rate for premalignant conditions, and use of artificial intelligence.

### 5.3 Discussion

This study employs a unique methodology to take into account patient perspectives in addition to the current evidence and experts' opinion, in order to identify the KPIs which can improve endoscopy quality and reduce PEUGIC. Up to three‐fold variations in PEUGIC rates has been observed among endoscopy providers in England, highlighting the need for standardisation of UGI endoscopy practices [3]. Adoption of the proposed recommendations can contribute to minimising the variation among providers.

Monitoring of PEUGIC rates and less intense endoscopy lists were considered the most important provider level KPIs. The BSG and JAG recommend that endoscopy providers should audit their PEUGIC rates and aim to keep PEUGIC rates below 10% [4]. However, our recent analysis of NCRAS and HES databases for all UGI cancers diagnosed between 2009–2018 in England reported that the national PEUGIC rate was 8.5%, with the 25th centile rate 7% [3]. It was estimated that if the national PEUGIC rate can be reduced to the 25th centile rate, there would be 162 fewer patients with PEUGIC each year. A recent multicentre root cause analysis of PEUGIC cases reported that up to 70% of PEUGIC cases were potentially avoidable [11]. Based on these reports, the panel agreed with a new benchmark rate of 7%. Some patients may be diagnosed with UGI cancer at a provider different from the provider where they had their initial endoscopy. Therefore, relying on local data will underestimate the PEUGIC rate. In order to overcome this, a national reporting system is being developed in England. PEUGIC will be identified nationally and providers where the patient had an endoscopy within the 3 years before diagnosis alerted. Providers will then perform root cause analysis to identify missed opportunities in potentially avoidable cases.

Endoscopy providers should ensure that enough time for UGI endoscopy is allocated to allow high‐quality examinations. In the UK, endoscopy providers use a points system to allocate time for procedures. Although there is provider‐level variation, in general, 15–20 min are assigned for one point and an UGI endoscopy or flexible sigmoidoscopy is allocated one point and a colonoscopy two points. In the NED analysis, a negative association was found between more intense endoscopy lists (average points per list > 9) and the best PEUGIC rate providers [12].

In the UK, the JAG provides external accreditation of endoscopy providers. This is a quality assurance program, and providers are assessed against a set of standards covering four domains: clinical quality, patient experience, clinical workforce and training. Providers which did not participate in the JAG accreditation process and those which were assessed and required improvement had higher PEUGIC rates compared to those which were awarded and maintained JAG accreditation [3]. These findings suggest that external accreditation can improve the quality of endoscopy. In the UK, all endoscopy providers should aim to achieve and maintain JAG accreditation.

Premalignant conditions (including Barrett's oesophagus, gastric atrophy and gastric intestinal metaplasia) were strongly associated with PEUGIC [3]. Retrospective studies have shown that the diagnostic yield of dysplasia was higher if Barrett's surveillance was performed by endoscopists trained in its assessment and on dedicated lists where extra time was allocated [13, 14, 15]. Although no data is available on the impact of dedicated lists on dysplasia detection for other premalignant conditions (e.g. gastric atrophy and intestinal metaplasia), the panel recommended that similar expertise and dedicated lists were required for such surveillance endoscopy.

Examination time is an important operator dependent aspect of endoscopy. A multicentre study reported that lengthening the Barrett's inspection time (> 1 min/cm) resulted in better diagnostic yield for suspicious lesions [13]. Three other studies have reported that longer examination times were associated with higher neoplasia detection rates [16, 17, 18]. Kwamura et al. examined data for 15,763 screening endoscopy and reported that the UGI cancer detection rates were higher for endoscopists with average examination times over 7 min [18]. Another retrospective study reported that examination times over 7 min were associated with a 3‐fold increased likelihood of detection of gastric cancer [17]. It was agreed that an UGI endoscopy should take a minimum of 7 min [4, 5].

Currently, the JAG endoscopy curriculum in the UK is focussed on acquiring technical skills with insufficient emphasis on training in the identification of early UGI neoplastic changes. A structured learning programme can increase the knowledge and recognition skills of endoscopists to detect early UGI cancer. A randomised controlled trial that enroled 335 endoscopists from 35 countries around the world investigated the value of an e‐learning programme to detect early gastric cancer and found higher test scores in the e‐learning group compared to the control group [19]. An observational study from China reported that a lesion recognition training programme in a real world clinical setting resulted in increased detection of early gastric cancer [20]. Structured training programmes and summative assessments based on an endoscopy curriculum should therefore be introduced to improve the detection and characterisation of pre‐malignant and early malignant changes. It is currently challenging to monitor early neoplasia detection rates for endoscopists, but in future, once systems are in place which can link endoscopy reporting systems with the histological diagnosis, it will be possible to monitor neoplasia detection rates at UGI endoscopy, which will be key to efforts to improve lesion recognition.

Defoaming (e.g. Simethicone) and mucolytic agents (e.g. N‐Acetylcysteine and Pronase) have been investigated in randomised controlled trials [21, 22, 23, 24, 25, 26]. The use of Simethicone, in combination or alone, provides significantly better mucosal views [21, 22, 23, 24]. N‐Acetylcysteine did not improve mucosal visibility if Simethicone was given beforehand [22, 27], although a significantly smaller number of patients required flushing during endoscopy. A randomised controlled trial from South Korea reported that pronase can improve mucosal visibility and the negative predictive value of NBI at endoscopy [28]. Recently, a scoring system has been proposed to assess mucosal cleanliness during UGI endoscopy and it was found to be associated with a higher detection of clinically significant lesions [29]. The consensus panel agreed that mucosal cleansing techniques should be routinely used to achieve good mucosal views.

In the NED analysis, it was noted that the use of intravenous sedation was more common among providers with the best PEUGIC rates [12]. Concomitant use of opioids and benzodiazepines was also more common among providers with the lowest PEUGIC rates. A multicentre study from China reported higher UGI cancer and high grade dysplasia detection rates with Propofol [30]. These observations are likely related to better patient tolerance with sedation, allowing adequate examination time and use of image enhancement techniques as required. Where appropriate, considering patient factors, intravenous sedation should be offered to all patients undergoing UGI endoscopy. The panel was not able to recommend a minimum standard for use of IV sedation, but the rate, benchmarked against national data, can be used as an auditable outcome.

In the NCRAS analysis, premalignant (e.g. Barrett's oesophagus) and cancer associated lesions (oesophageal ulcer/stricture, severe oesophagitis and gastric ulcer) were found to be strongly associated with PEUGIC [3]. Inadequate assessment of high risk lesions and decision making on surveillance or follow‐up was one of the most common reasons for missing the cancers on PEUGIC root cause analysis [11]. National recommendations e.g. from the BSG should be followed when assessing these lesions and endoscopy providers should audit compliance with these recommendations [4, 31, 32].

The BSG proposes that the endoscopists should aim to perform a minimum of 100 procedures annually to maintain their proficiency and endoscopy providers are required to monitor the annual volume of UGI endoscopy performed by endoscopists [4, 33]. A NED analysis reported that around half of the endoscopists in the UK met the 100 UGI endoscopy per year minimum recommended volume [34]. A multicentre root cause analysis of PEUGIC described a negative correlation between annual UGI endoscopy volume and the PEUGIC rates of individual endoscopists [11]. In a recent NED analysis, a positive association was found between annual endoscopist volume > 100 and providers with the best PEUGIC rates, supporting the BSG recommendation [12].

Provider level KPIs offer granular insights that are essential for detailed performance assessment and can be used to compare the performance of different healthcare providers. For the first time, intensity of endoscopy lists, external accreditation and dedicated lists for surveillance were proposed as KPIs to assess the efficacy and quality of services provided. A new benchmark was also proposed for the PEUGIC rate. These KPIs can be instrumental in identifying best practices and areas of improvement when comparing different providers.

Endoscopy practices vary across the country. This provides an opportunity to compare the effect of various interventions suggested in this consensus on endoscopy quality. Future work, including controlled trials with cluster randomisation of endoscopy providers to test a bundle of interventions (e.g. training endoscopists in lesion recognition, dedicated lists for surveillance and follow up endoscopy of high risk conditions, use of premedications, less intense endoscopy lists and minimum examination time more than 7 min) and compare it with standard care, should be considered to establish the impact of these interventions on cancer and dysplasia detection rates.

This study has a number of limitations. Due to the lack of well‐designed trials, most of the evidence examined was graded as low to moderate quality. As the group of experts and participating patients were all from the UK, there is a potential bias that these recommendations may be more applicable to UK endoscopy practice. We expect that the current study provides a framework for further research in other healthcare systems to corroborate or refute our recommendations. Due to the paucity of evidence in the field, it was not possible to suggest minimum standards for some measures. However, assessment of those KPIs was considered important. Such KPIs are sometimes described as ‘auditable outcomes’ and it is hoped that future research will help determine appropriate standards [35]. We recognise that bias may be evident in consensus statements receiving higher ranking, as major KPIs with only weak evidence. We allowed a discordantly high ranking when the consensus group determined that a particular KPI was integral to assessing the quality of endoscopy. While this leaves these recommendations open to criticism, we hope that by setting these standards, high‐quality research can be undertaken to validate these findings.

## 6 Conclusions

This consensus provides a list of major and minor KPIs to improve the quality of endoscopy and reduce PEUGIC. This framework will enable endoscopy providers to monitor their performance and ensure the provision of a high‐quality UGI endoscopy service for their patients.

## Author Contributions

Study concept and design was jointly conceived by UK and NT. Consensus meetings were co‐ordinated by UK, NT and TAG. The manuscript was draughted by UK. The manuscript was critically reviewed, revised, and approved by all authors.

## Ethics Statement

The study was approved by the London ‐ South East Research Ethics Committee (IRAS project ID # 289695).

## Conflicts of Interest

The authors declare no conflicts of interest.

## Supporting information

Supporting Information S1

## Data Availability

The data that support the findings of this study are available on request from the corresponding author. The data are not publicly available due to privacy or ethical restrictions.
