# Supplementary material for: Consensus on Upper Gastrointestinal Endoscopy Key Performance Indicators to Reduce Post Endoscopy Upper Gastrointestinal Cancer
Source: United European Gastroenterol J. 2025 Jul 24;13(8):1438–45. doi: 10.1002/ueg2.70001 (PMC12529009; doi:10.1002/ueg2.70001)
Supplement: Supplementary file 1 — Supporting Information S1 [file UEG2-13-1438-s001.docx]

**Supplementary table 1 Individual group and mean ranking scores for the key performance indicators for upper gastrointestinal endoscopy**

| **Key performance indicators** | **Group 1** | **Group 2** | **Group 3** | **Geometric mean** |
| --- | --- | --- | --- | --- |
| **Endoscopy provider related** |  |  |  |  |
| 1. Monitoring of endoscopy provider PEUGIC rate | 1 | 4 | 2 | 2.0 |
| 2. Less intense endoscopy lists | 3 | 1 | 3 | 2.1 |
| 3. External accreditation of endoscopy providers | 4 | 3 | 1 | 2.3 |
| 4. Surveillance of high-risk conditions performed on dedicated lists by endoscopists with adequate training | 2 | 2 | 4 | 2.5 |
| **Endoscopist or procedure related** |  |  |  |  |
| *5. Minimum examination time ≥7 minutes | 2.5 | 1 | 3 | 2.0 |
| *6. Endoscopists should have dedicated training in recognition of early UGI neoplasia | 2.5 | 2 | 2 | 2.2 |
| *7. Mucosal cleaning agents used to achieve good mucosal views | 2.5 | 3 | 4 | 3.1 |
| *8. Intravenous sedation offered to all patients | 2.5 | 5 | 5 | 4.0 |
| **9. If a cancer associated lesion is identified, recommended number of biopsies taken | 8 | 10 | 1 | 4.3 |
| 10.  Minimum annual Upper Gastrointestinal endoscopy volume >100 | 5 | 4 | 6 | 4.9 |
| **11. If a premalignant lesion is identified, recommended number of biopsies taken | 6 | 7 | 7 | 6.6 |
| ***12.  Detection rate for premalignant conditions monitored | 7 | 6 | 9 | 7.2 |
| ***13. Detection rate for early dysplastic changes monitored | 10 | 11 | 8 | 9.6 |
| 14.  Image enhancement techniques used, especially in high-risk patients | 9 | 9 | 11 | 9.6 |
| 15.  Photo documentation of important anatomical sites | 11 | 8 | 12 | 10.2 |
| 16.  Neoplasia detection rate monitored | 14 | 13 | 10 | 12.2 |
| 17.  Artificial intelligence | 13 | 14 | 14 | 13.7 |

PEUGIC - Post Endoscopy Upper Gastrointestinal Cancer

*In group 1, these four indicators were considered equally important and hence the score 2.5 was given to each indicator, which is mean of 1,2,3 and 4.

**Indicators combined in final statement

***Indicators combined in final statement
